# Fatigue Properties of Methacrylic Adhesive Plexus MA300

**DOI:** 10.3390/ma18092127

**Published:** 2025-05-06

**Authors:** Paweł Maćkowiak

**Affiliations:** Faculty of Mechanical Engineering, University of Science and Technology, 85-796 Bydgoszcz, Poland; pawel.mackowiak@pbs.edu.pl

**Keywords:** methacrylic adhesive fatigue, Plexus MA300, fatigue properties, high-cycle fatigue (HCF), energy dissipation analysis

## Abstract

This study investigates the fatigue durability of Plexus MA300 methacrylic adhesive, which is employed in structural joints of metals, plastics, and composites. Cast adhesive specimens were subjected to cyclic tensile loads at a frequency of 5 Hz with a stress ratio *R* = 0.1. Six load levels were tested. Hysteresis loops were recorded during testing and analyzed in detail. Significant differences in fatigue fracture characteristics were observed depending on load level. Specimens subjected to high loads exhibited a characteristic radial structure with a distinct crack initiation point, whereas specimens tested at lower loads showed more uniform, matte fracture surfaces. Hysteresis loop analysis revealed phenomena typical for polymers: creep and damping causing energy dissipation. Various fatigue approaches were compared: stress-based, strain-based, energy-based, and stiffness-based. The highest coefficient of determination (*R*²) was obtained for the model based on strain energy density, indicating its superior utility in predicting the fatigue life of the tested adhesive. The obtained results contribute to the understanding of the fatigue behavior of methacrylic adhesives and provide practical data for structural joint design involving this material class.

## 1. Introduction

In contemporary engineering structures, mass minimization is a primary objective. This is particularly crucial in transportation, where it reduces energy consumption during operation. Consequently, the use of materials with significantly different properties within a single object is common. These joints may involve similar materials, such as steel and aluminum. Alternatively, they may combine dissimilar materials, such as steel and plastics. Among the many joining techniques, structural bonding is commonly chosen. Bonding offers several advantages, including reduced stress concentration, simplified and rapid assembly, and the ability to seal structures. However, bonded joints typically exhibit lower resistance to environmental conditions and reduced adhesive strength compared to the joined materials [1,2,3].

The mechanical properties of the adhesive as a material are crucial when calculating the strength and durability of the joints it creates [4,5,6]. Most material tests have focused on epoxy adhesives. These adhesives have been tested for both their strength [7,8] and fatigue durability [9,10]. Significantly less research has concerned the properties of methacrylate adhesives. Most of these studies focus on the properties derived from monotonic tensile tests [11,12]. Previous research indicates differences in mechanical properties between methacrylate and epoxy adhesives [13]. Fatigue durability tests of cast methacrylate adhesive samples are conducted much less frequently. One of the few examples of such research is the manufacturer’s research [14]. Research on joints made with methacrylate adhesives has shown that their elastoplastic properties lead to ductile failure, which is beneficial for large lap joints of high-strength steel [15,16].

For cohesive failure in adhesive joints, the strength can be correlated to the adhesive’s material properties. However, this relationship is complex and influenced by several factors [17,18]. Calculation methods for bonded joints should be comparable in complexity to those for welded joints, such as fatigue strength classes (FAT classes), the hot-spot method, and the effective notch stress method [19,20]. However, the stress state in a bonded joint is more complex and depends on many factors such as the mechanical properties of the joined materials, the adhesive, and the geometry of the joint itself [21,22]. The stress state of the weld is also complex, which complicates the analysis as it is necessary to choose appropriate strength hypotheses [23,24].

The methods used to predict the strength and durability of bonded joints can be categorized by their failure criteria:

- Maximum stress criterion [25,26] or maximum strain criterion [27,28];
- Critical stress criterion [29,30] or critical strain criterion at a distance or over an area [31,32];
- Limit state criterion [33,34];
- Fracture mechanics criteria [35,36].

These methods require knowledge of the adhesive’s mechanical properties, which are used in analytical and numerical calculations. The level of complexity of the calculations often exceeds the engineering possibilities of their application [5]. The relatively simplest methods are based on stress–strain and S-N curves. It is most advantageous when all experimental results can be reduced to one master curve. This can be achieved by using an effective fatigue parameter *σ_eff_*, determined through analytical and numerical methods. Furthermore, *σ_eff_* can be a combination of several factors, including stresses, strains, and many others [37].

The increasing use of methacrylate adhesives in engineering structures under cyclic loading makes their fatigue analysis a relevant and important research area. In contrast to the extensively studied fatigue characteristics of epoxy adhesives [9,10], the fatigue characteristics of methacrylates are still relatively poorly documented in the scientific literature. This is attributed to two factors. First, methacrylate adhesives have seen widespread use more recently than epoxy adhesives. Second, methacrylate adhesives and their joints exhibit different microstructures, mechanical properties, and fracture mechanisms compared to epoxy adhesives [12,13]. Understanding the behavior of methacrylate adhesives under variable loads is crucial for ensuring the durability and safety of structures in which they are used.

The aim of this work is to determine the durability of the methacrylate adhesive Plexus MA300. The manufacturer of the adhesive used in this study was ITW Performance Polymers, located in Danvers, MA, USA. While the author’s previous work has focused on the static mechanical properties of Plexus MA300 methacrylic adhesive [12,13,38,39], a significant gap exists in the understanding of its fatigue behavior. This study aims to address this gap by thoroughly investigating the fatigue durability of this adhesive under cyclic tensile loading. Additionally, in order to select the best-fitting model for the test results, fatigue approaches other than stress-based ones were verified, such as an energy-based approach, accumulated energy approach, strain-based approach, and dynamic stiffness approach. The determined properties of the adhesive will be used in the author’s future work, the aim of which will be to verify selected calculation methods for the fatigue durability of bonded joints made using methacrylate adhesive.

## 2. Materials and Methods

### 2.1. Materials

**Figure 1:**
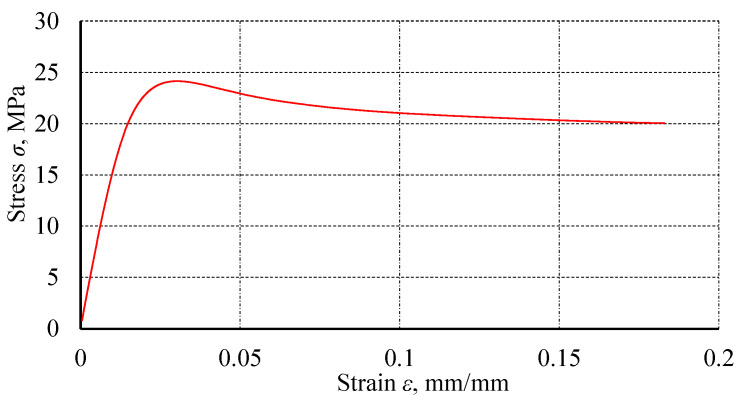
An exemplary graph from the monotonic tensile test of the methacrylate adhesive Plexus MA300.

Plexus MA300 methacrylate adhesive was used for testing. Table 1 presents the mechanical properties of the methacrylate adhesive, determined using a monotonic tensile test, as detailed in a previous publication [13]. The specimen was tested at a constant displacement rate of 0.05 mm/s. Figure 1 shows a graph detailing an exemplary specimen.

### 2.2. Methods

Methacrylic adhesive specimens were prepared from cast plates with dimensions of 75 mm × 45 mm × 5 mm. A mixer, recommended by ITW Performance Polymers, was used for mixing two components of the adhesive. The adhesive paste was applied to the mold, which was then closed at a slight angle to ensure even distribution. This method of application reduces the risk of air bubbles in the casting. After curing, the cast plates of the methacrylic adhesive were removed from the mold. Then, the target shape of the specimen was milled from the plate. This method of producing cast plates from methacrylic adhesive was described in a previous work [12].

**Figure 2:**
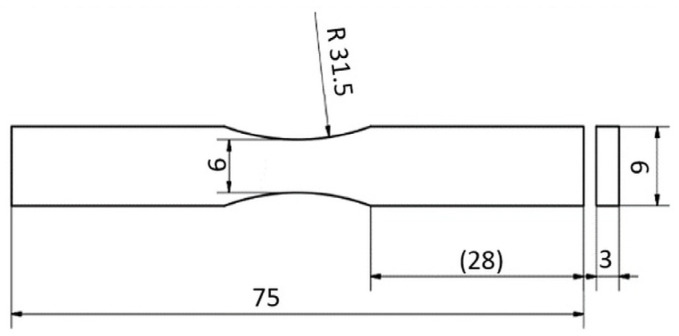
The dimensions of the specimens used for tests under cyclic load conditions (all values are given in millimeters).

**Figure 3:**
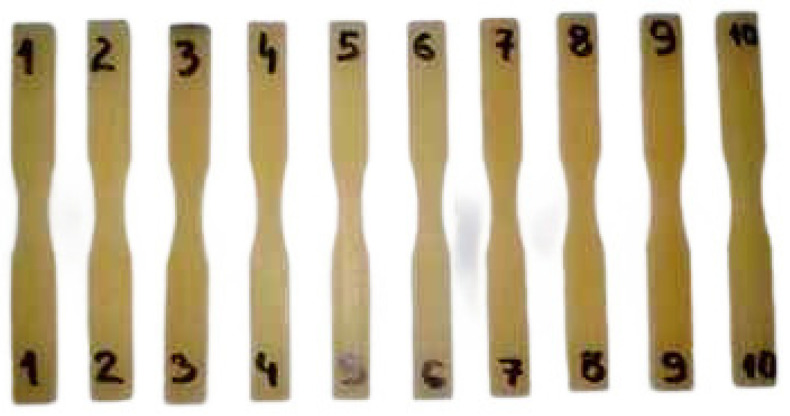
The specimens used for tests under cyclic load conditions.

Plexus MA300 adhesive specimens were prepared according to the dimensions in Figure 2, with an example shown in Figure 3. Specimens were conditioned for one month at room temperature (temperature 20 ± 2 °C, humidity 45 ± 5%).

**Figure 4:**
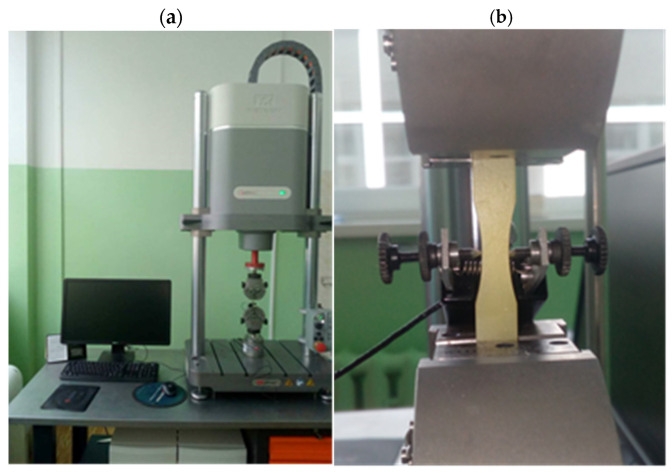
The test stand used for fatigue tests of methacrylate adhesive: (**a**) the Instron E3000 testing machine; (**b**) a specimen mounted in the machine’s grips, with an extensometer for measuring transverse strain.

The fatigue durability test of the Plexus MA300 methacrylate adhesive was conducted using an Instron E3000 testing machine (Figure 4a). The cycle asymmetry ratio *R* = 0.1 was used. The tests were carried out under force control, varying sinusoidally with a frequency of *f* = 5 Hz. Data were recorded at 1000 Hz, capturing 200 points per load cycle. An extensometer for measuring transverse strain was mounted on the specimens (Figure 4b).

Each test began with a 5-s ramp to the minimum force value. Then, the program proceeded to cyclic loading. The maximum load in the cycle *σ*_max_ was calculated as a percentage of the material’s tensile strength from static tests:

(1) $$\sigma_{max}=\sigma_{u}\cdot X$$

where:

*σ_u_*—tensile strength;
*X*—percentage value.

The minimum load in the cycle *σ*_min_ was calculated from the following relationship:

(2) $$\sigma_{min}=0.1\;\sigma_{max}$$

An analysis of the literature data and previous own self-conducted research have shows shown that the fatigue properties of the adhesive are not correlated with their properties determined under static loading conditions. Several factors influence this, in-cluding the strain rate, which is significantly lower in the case of static tests. The results of polymer tests show that the tensile strength can increase even by 10 times with an in-crease in the strain rate [42]. A higher strain rate limits changes in the polymer structure and, as a result, increases its strength properties [43]. Therefore, in this work, it was de-cided to determine the fatigue properties of the tested adhesive, taking into account load levels corresponding to static strength, as well as those exceeding it. Six load levels were used: 110% σu, 100% σu, 90% σu, 80% σu, 70% σu, 60% σu. Table 2 summarizes the maximum and minimum stress values in the cycle. In addition, the stress amplitude σa and the mean stress σm are given.

Specimen force values were calculated by multiplying the stress by the specimen’s cross-sectional area, measured at the gauge section’s narrowest point before testing.

## 3. Results

Table 3 presents the fatigue test results for the Plexus MA300 methacrylate adhesive, including maximum stress (*σ*_max_), stress amplitude (*σ_a_*), and cycles to failure (*N*), arranged from the highest to lowest load levels, the stress amplitude *σ_a_*, and the number of cycles to failure *N*. Thirty-six specimens were tested. Two specimens tested at the lowest load level did not fail after 5 million cycles. Above this value, the test was stopped.

**Figure 5:**
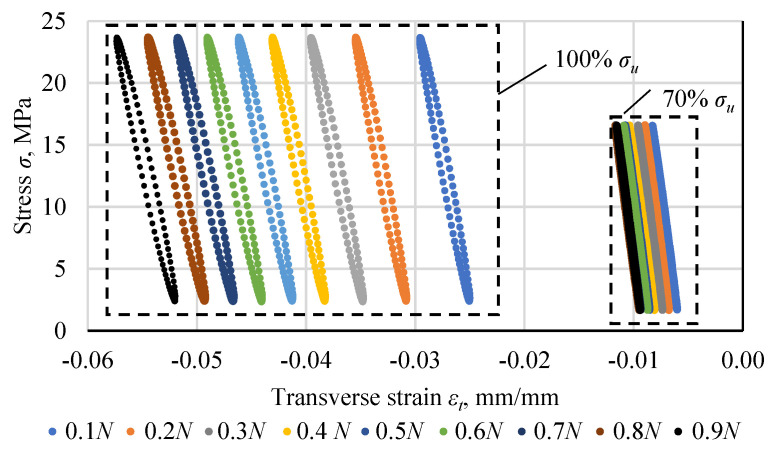
Hysteresis loops for the cycles representing 0.1 to 0.9 of the fatigue life of the Plexus MA300 methacrylate adhesive specimens loaded with a maximum stress of 100% *σ_u_* and 70% *σ_u_*.

**Figure 6:**
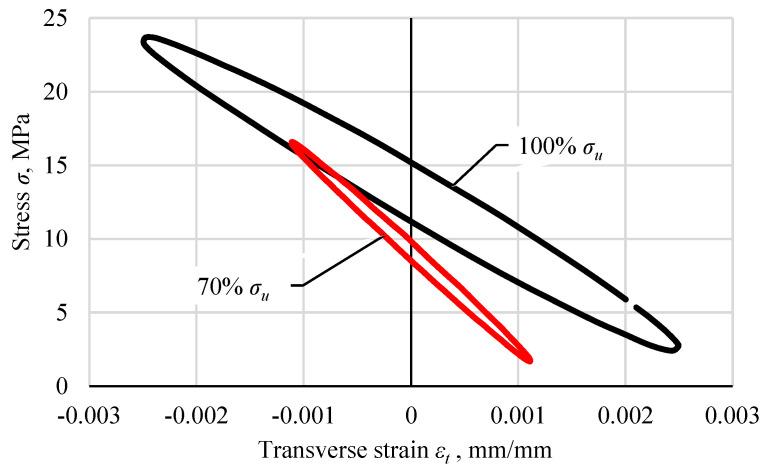
An exemplary comparison of the hysteresis loops for the cycles representing 0.5 of the fatigue life of the Plexus MA300 methacrylate adhesive specimens loaded with a maximum stress of 100% *σ_u_* and 70% *σ_u_*.

During the tests, stresses and strains were recorded in each cycle. Figure 5 shows hysteresis loops for a specimen loaded with a maximum stress in the cycle at 100% *σ_u_* (*σ*_max_ = 23.7 MPa) and 70% *σ_u_* (*σ*_max_ = 16.6 MPa). The first one reached 34,016 cycles, the second one 1,277,175 cycles. The graphs show selected hysteresis loops in the range from 0.1 *N* to 0.9 *N*, every 0.1 *N*. Figure 6 shows hysteresis loops from half the fatigue life of both exemplary specimens. The loops were shifted to the origin by subtracting the strain value resulting from cyclic creep.

## 4. Discussion

**Figure 7:**
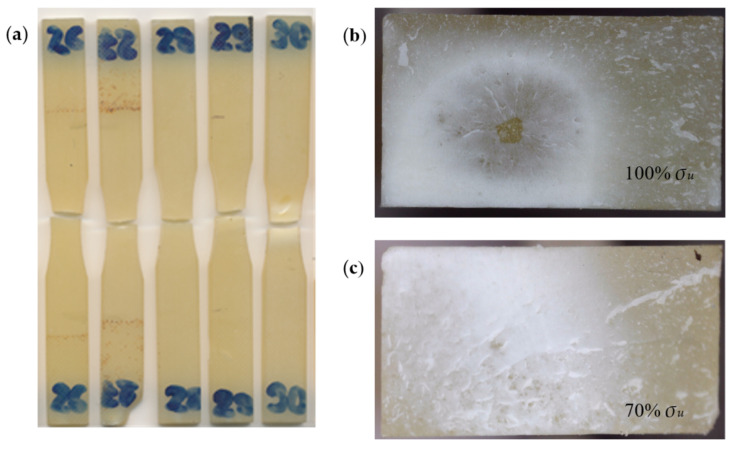
The specimens after testing: (**a**) exemplary specimens; (**b**) a fracture of a specimen loaded at 100% *σ_u_*; (**c**) a fracture of a specimen loaded at 70% *σ_u_*.

Figure 7 shows representative images of fractured specimens. Fractures corresponding to the specimens in Figure 5 are shown in Figure 7b,c. In polymeric materials, fatigue fractures result from the progressive accumulation of microdamage under cyclic loading, leading to macroscopic failure. On the fracture surface of the 100% *σ_u_*-loaded specimen (Figure 7b), a characteristic crack initiation zone (central point) and a crack propagation zone (radially spreading structure) are visible. The tested specimen shows features typical of a fatigue fracture of a polymeric material subjected to loads close to its ultimate strength. These characteristics include the following: a clear crack initiation site at the specimen’s center, a radial crack propagation pattern (river pattern), and a relatively smooth fracture surface with limited plastic deformation (indicated by color change to white) [44]. At loads near the ultimate strength, as in this case, stress concentration induces polymer chain scission. Additionally, the failure mechanism is related to heat build-up as a result of mechanical hysteresis of the polymeric material during cyclic loading [45,46]. The second image (Figure 7c) shows a fatigue fracture of a specimen that was subjected to cyclic loading of a significantly lower value of 70% *σ_u_*, withstanding approximately one million cycles before failure. This fracture surface exhibits a distinctly different morphology compared to the previous one. The surface is smoother and more uniform, lacking a clear, singular crack initiation point. Small irregularities are visible over the entire fracture surface, and the radial crack propagation pattern is less pronounced and runs from the edge to the center of the specimen. The lighter, matte surface indicates a slower crack propagation process. The morphology is characteristic of high cycle fatigue (HCF), where the material fails after a large number of cycles at relatively low stress levels. In the case of high cycle fatigue of acrylic polymers, the failure process includes the formation of micropores and microcracks dispersed through a larger volume of the material; the slow coalescence of microcracks into larger structures; and gradual degradation of the polymer structure without a clear initiation point [46]. A smaller contribution of thermal effects to the failure process is also observed in this case. In polymers, at lower stress levels fatigue is dominated by microcrack accumulation processes, rather than by a single crack propagating from a clear initiation site [47,48].

**Figure 8:**
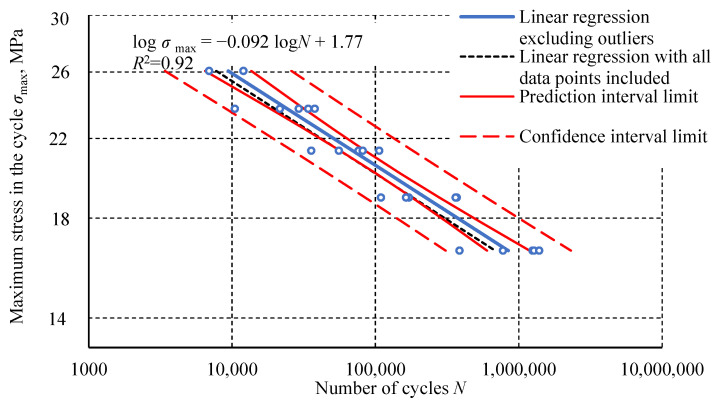
A fatigue life diagram (S-N curve) of the Plexus MA300 methacrylate adhesive specimens after outlier removal.

Outliers were removed using Student’s t-test with a significance level of α = 0.05. The final dataset comprised 24 specimens: 6 each for 100% *σ_u_* and 80% *σ_u_*, 5 each for 90% *σ_u_* and 70% *σ_u_*, and 2 for 110% *σ_u_*. These results are shown in the fatigue life diagram (Figure 8), with linear regression fitted to the data.

**Figure 9:**
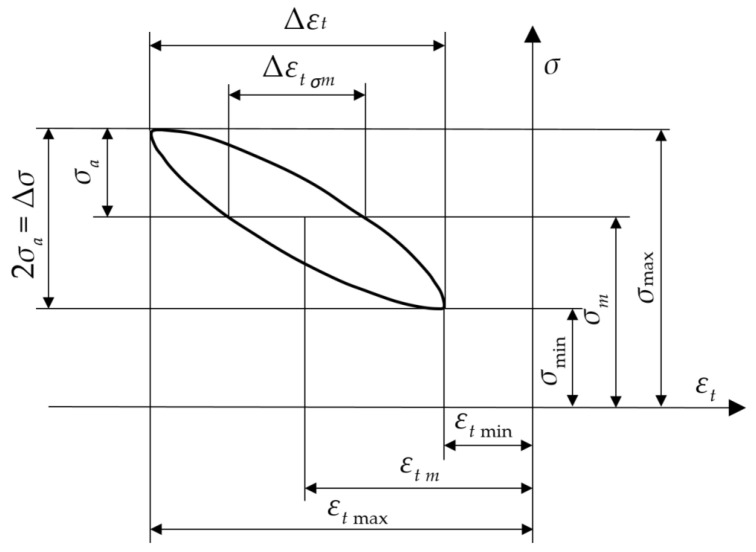
A diagram illustrating the parameters of the hysteresis loops.

Hysteresis loops were recorded and analyzed during the fatigue tests. Figure 9 illustrates the parameters of the hysteresis loops.

**Figure 10:**
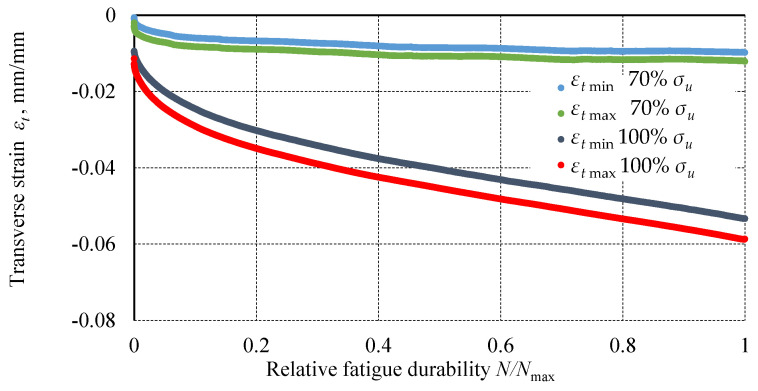
The transverse strains of the specimens as a function of relative fatigue life for the 100% *σ_u_* and 70% *σ_u_* levels.

Analysis of fatigue test results at various load levels (100% *σ_u_*, 90% *σ_u_*, 80% *σ_u_*, 70% *σ_u_*) showed that the maximum transverse strain *ε_t_*_max_, the transverse strain range of the cycle Δ*ε_t_*, and the transverse strain range at mean stress Δ*ε_tm_* increased with the number of cycles. These transverse strain ranges are plotted against relative fatigue life in Figure 10.

The strains in the specimens loaded at higher levels are greater and their values increase faster than in the specimens loaded at lower levels. The center of successive hysteresis loops shifts with the number of cycles, which is characteristic of creep in plastics. In many materials, particularly amorphous plastics, strain can increase under constant load [46]. This is due to the straightening and movement of polymer chains relative to each other under load. Creep is characterized by three stages: primary creep, with a decreasing strain rate; secondary creep, with a constant strain rate; and tertiary creep, with an increasing strain rate. The graphs show primary and secondary creep. However, tertiary creep is not observed.

These observations on creep and viscoelastic properties are characteristic features of methacrylate adhesives, as confirmed in many studies [43,49]. For example, in the work of Singh et al. [49], these phenomena were studied in detail using model methacrylate adhesives for dentine. Creep tests and static tests were performed at different loading rates, which allowed the development of a linear viscoelastic model describing the adhesives’ behavior, including the speed-dependent modulus of elasticity. This model was then used to interpret the results of fatigue tests, emphasizing that viscoelastic phenomena have a significant influence on the behavior of the material under cyclic loading and may contribute to its failure.

**Figure 11:**
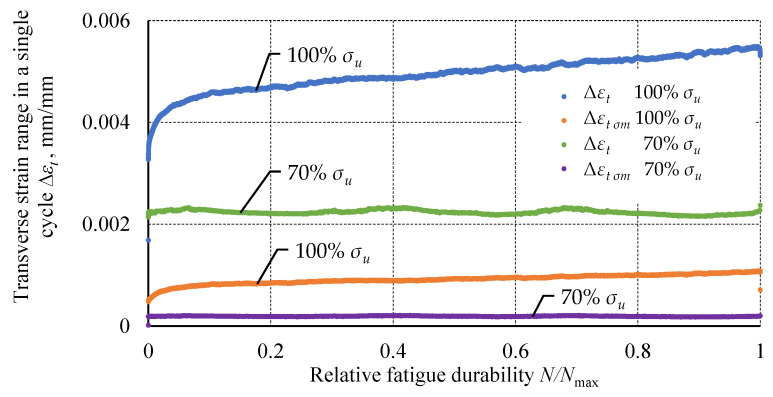
A comparison of strains Δ*ε_t_* and Δ*ε_t σm_* at load levels of 100% *σ_u_* and 70% *σ_u_* as a function of relative fatigue life.

The below graph (Figure 11) shows changes in the ranges Δ*ε_t_* and Δ*ε_t σm_* for specimens loaded at 100% *σ_u_* and 70% *σ_u_*. For the specimens loaded at the higher level, an increase in these ranges with an increasing number of cycles can be observed. A faster increase occurs for the total transverse strain in the cycle. The indicated parameters have an observable transient and steady-state stage in terms of the strain range increase rate. The specimens loaded at 70% *σ_u_* show different behavior. Transverse strain ranges remain constant. The slight variability present in the graph may be due to external factors disturbing the measurement. However, no trend is observed (slope of linear regression *a* ≈ 0). The strain ranges are more than two times smaller than the strain ranges of the specimens loaded at 100% *σ_u_*.

**Figure 12:**
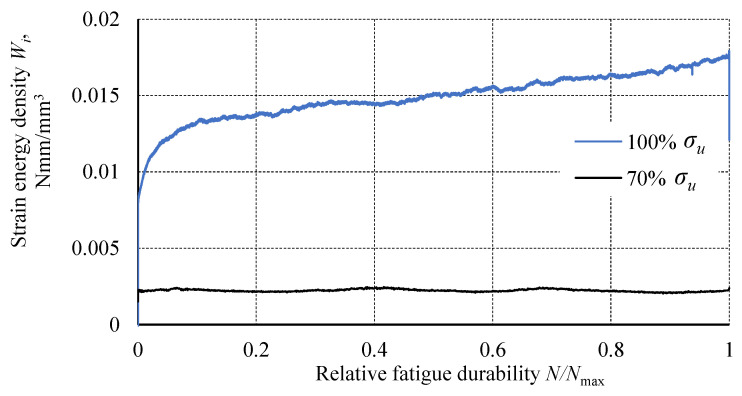
Dependence of strain energy density as a function of relative fatigue life for load levels 100% *σ_u_* and 70% *σ_u_*.

The strain energy density, represented by the area within each hysteresis loop, was calculated. This value increased with the number of cycles for the 100% *σ_u_* specimen, while remaining constant for the 70% *σ_u_* specimen. Comparing the indicated results (Figure 12), it can be observed that the values of this parameter are several times greater for the specimen tested at the 100% *σ_u_* load level. The value of the strain energy density is not proportional to the load level of the specimen.

The analysis of hysteresis loops allows for the application of a strain-based approach (log*ε_tσm_* − log*N*), an energy-based approach (log*W_i_* − log*N*), and a stiffness-based approach (log*E_d_* − log*N*). The stiffness-based approach involves determining the dynamic stiffness modulus based on the data from the loops. The secant dynamic modulus defined by the following formula was adopted:

(3) $$E_{d}=\frac{\sigma_{max}-\sigma_{min}}{∆\varepsilon_{t}}$$

**Figure 13:**
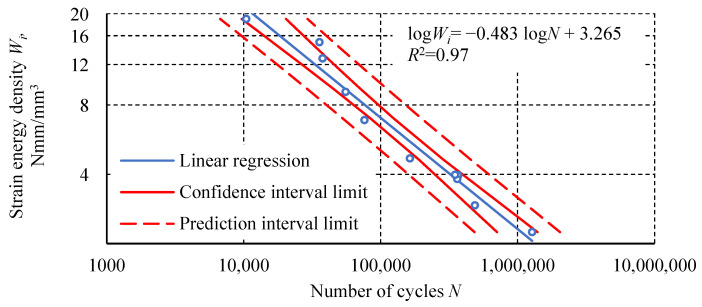
The dependence of strain energy density *W_i_* as a function of the number of cycles.

**Figure 14:**
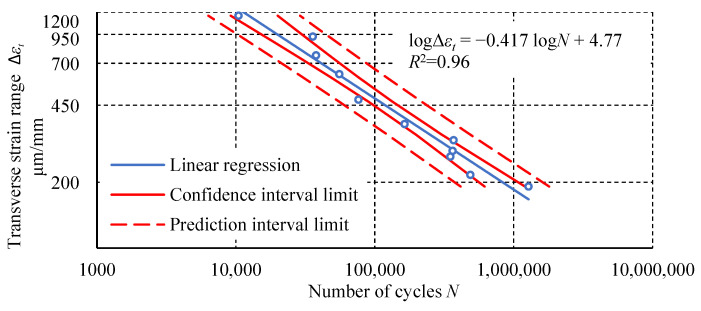
The dependence of strain Δ*ε_t_* at half the fatigue life of the specimen as a function of the number of cycles.

**Figure 15:**
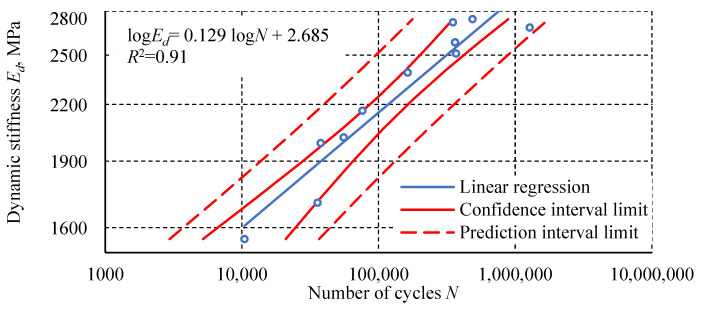
The dependence of the dynamic stiffness of specimen *E_d_* as a function of the number of cycles.

The following graphs (Figure 13, Figure 14, Figure 15) show the measurement results of the loop analysis in the form of the dependence of selected parameters on the number of cycles. For each graph, a statistical analysis was performed, which involved determining linear regression and the coefficient of determination *R*^2^. In addition, confidence intervals for linear regression and prediction intervals for α = 0.05 were determined.

**Figure 16:**
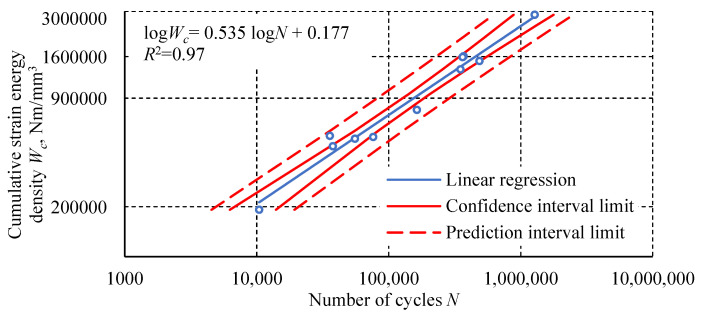
The dependence of cumulative strain energy density *W_c_* as a function of the number of cycles.

In addition to the analysis characterizing individual loops, cumulative values can also be considered. These values are the sum of the parameters determined for all individual hysteresis loops up to the failure of a given specimen. One of them is the cumulative strain energy density as a function of the number of cycles (Figure 16). This parameter defines the energy dissipated in all cycles until the specimen fails.

**Figure 17:**
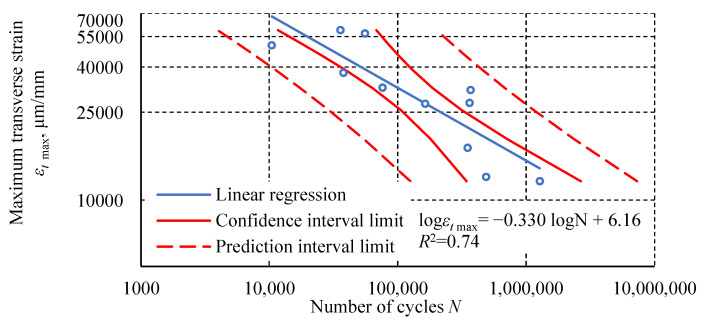
The dependence of the maximum transverse strain *ε_t_*_max_ in the last cycle before failure as a function of the number of cycles.

The dependence of the maximum transverse strain in the last full cycle before failure as a function of the number of cycles was characterized by a lower linear regression fit (Figure 17). In the graph, the value of this parameter is presented as the strain increment from the beginning of the loading of the specimen. A log-log scale was used for all the discussed dependencies and graphs. In each approach discussed in this section, a semi-logarithmic scale resulted in a lower fit of the linear model to the obtained results.

## 5. Conclusions

The fatigue test results of the Plexus MA300 methacrylate adhesive are characterized by significant scatter. Statistical outlier removal increases the coefficient of determination of the simple regression, with minimal effect on its slope and intercept. Hysteresis loop analysis revealed phenomena characteristic of plastics. These phenomena include creep and damping, which cause energy dissipation. These phenomena result from polymer chain uncoiling in the amorphous phase and interchain friction. The final stage of transient creep, characterized by an increasing strain rate, is not observed in the fatigue-tested adhesive specimens. This may indicate that creep is not the main cause of specimen failure. Specimens tested at higher load levels show a distinctly higher steady-state creep rate. Specimens loaded at 100% *σ_u_* exhibit an increased transverse strain range and hysteresis loop width at mean stresses. For specimens tested at the lowest level, these parameters have a constant value or stabilize after the first few cycles. The diagram of fatigue life in terms of strain energy density is characterized by a higher coefficient of determination *R*^2^ for the regression line than the diagram of fatigue life in terms of stress. Higher *R*^2^ values were also obtained for the regression lines relating to an average transverse strain Δ*ε_t σm_* and cumulative strain energy density compared to the number of cycles. The regression line for the dependence of dynamic stiffness on the number of cycles achieved a similar *R*^2^ value to the regression line of the diagram in terms of stress. The relationship between the maximum transverse strain *ε_t_* _max_ in the last cycle before failure and the number of cycles shows the poorest fit of the regression line to the experimental results.

## Figures and Tables

**Table 1:** The adhesive properties specified by the manufacturer and experimental data.

Adhesive Properties	Tensile Strength	Young’s Modulus *E*	Strain to Failure	Lap Shear (ASTM D1002) [40]
	MPa	MPa	%	MPa
Data from adhesive manufacturer [41]	20 to 24	931 to 1137	15 to 25	15 to 25
Experimental data [13]	23.7	1610.5	19,4	21.1

**Table 2:** The stress values in the cycle for specific load levels.

Load Levels	Stress Values in the Cycle			
	*σ*_max_	*σ*_min_	*σ_a_*	*σ_m_*
110% *σ_u_*	26.1	2.61	11.7	14.4
100% *σ_u_*	23.7	2.37	10.7	13.0
90% *σ_u_*	21.3	2.13	9.59	11.7
80% *σ_u_*	19.0	1.90	8.55	10.5
70% *σ_u_*	16.6	1.66	7.47	9.13
60% *σ_u_*	14.2	1.42	6.39	7.81

**Table 3:** The fatigue test results of the Plexus MA300 methacrylate adhesive.

Lp.	Maximum Stress in the Cycle *σ*_max_	Stress Amplitude *σ*_a_	Number of Cycles to Failure *N*	Lp.	Maximum Stress in the Cycle *σ*_max_	Stress Amplitude *σ_a_*	Number of Cycles to Failure *N*
-	MPa	MPa	-	-	MPa	MPa	-
1	26.1	11.7	12,010	19	21.3	9.6	106,262
2	26.1	11.7	6911	20	19.0	8.55	171,044
3	23.7	10.7	10,444	21	19.0	8.55	46,114
4	23.7	10.7	37,759	22	19.0	8.55	109,408
5	23.7	10.7	34,016	23	19.0	8.55	163,608
6	23.7	10.7	29,185	24	19.0	8.55	54,536
7	23.7	10.7	1187	25	19.0	8.55	369,123
8	23.7	10.7	37,726	26	19.0	8.55	487,583
9	23.7	10.7	21,585	27	19.0	8.55	362,728
10	21.3	9.59	76,298	28	19.0	8.55	163,635
11	21.3	9.59	11,169	29	16.6	7.47	1,248,717
12	21.3	9.59	319,102	30	16.6	7.47	1,277,175
13	21.3	9.59	350,025	31	16.6	7.47	190,599
14	21.3	9.59	55,540	32	16.6	7.47	775,502
15	21.3	9.59	6670	33	16.6	7.47	386,391
16	21.3	9.59	35,672	34	16.6	7.47	1,388,402
17	21.3	9.59	81,560	35	14.2	6.39	>5,000,000 *
18	21.3	9.59	156,890	36	14.2	6.39	>5,000,000 *

^*^ Specimen did not break, test stopped at 5,000,000 cycles.

## Data Availability

The original contributions presented in this study are included in the article. Further inquiries can be directed to the corresponding author.
